# The strength of the association between heterozygosity and probability of interannual local recruitment increases with environmental harshness in blue tits

**DOI:** 10.1002/ece3.2591

**Published:** 2016-11-21

**Authors:** Esperanza S. Ferrer, Vicente García‐Navas, Juan José Sanz, Joaquín Ortego

**Affiliations:** ^1^ Grupo de Investigación de la Biodiversidad Genética y CulturalInstituto de Investigación en Recursos Cinegéticos (CSIC‐UCLM‐JCCM)Ciudad RealSpain; ^2^ Departamento de Ciencias AmbientalesFacultad de Ciencias Ambientales y BioquímicaUniversidad de Castilla‐La ManchaToledoSpain; ^3^ Institute of Evolutionary Biology and Environmental StudiesUniversity of ZurichZurichSwitzerland; ^4^ Department of Integrative EcologyEstación Biológica de Doñana (EBD‐CSIC)SevilleSpain; ^5^ Departamento de Ecología EvolutivaMuseo Nacional de Ciencias Naturales (CSIC)MadridSpain

**Keywords:** *Cyanistes caeruleus*, functional and neutral markers, genotype‐by‐environment interaction, heterozygosity, HFC, selection differential

## Abstract

The extent of inbreeding depression and the magnitude of heterozygosity–fitness correlations (HFC) have been suggested to depend on the environmental context in which they are assayed, but little evidence is available for wild populations. We combine extensive molecular and capture–mark–recapture data from a blue tit (*Cyanistes caeruleus*) population to (1) analyze the relationship between heterozygosity and probability of interannual adult local recruitment and (2) test whether environmental stress imposed by physiologically suboptimal temperatures and rainfall influence the magnitude of HFC. To address these questions, we used two different arrays of microsatellite markers: 14 loci classified as neutral and 12 loci classified as putatively functional. We found significant relationships between heterozygosity and probability of interannual local recruitment that were most likely explained by variation in genomewide heterozygosity. The strength of the association between heterozygosity and probability of interannual local recruitment was positively associated with annual accumulated precipitation. Annual mean heterozygosity increased over time, which may have resulted from an overall positive selection on heterozygosity over the course of the study period. Finally, neutral and putatively functional loci showed similar trends, but the former had stronger effect sizes and seemed to better reflect genomewide heterozygosity. Overall, our results show that HFC can be context dependent, emphasizing the need to consider the role of environmental heterogeneity as a key factor when exploring the consequences of individual genetic diversity on fitness in natural populations.

## 1 Introduction

Inbreeding and reduced levels of genetic diversity have been found to negatively impact different components of fitness, including reproductive performance (Ortego et al., 2009; Seddon, Amos, Mulder, & Tobias, 2004), resistance to parasites (Acevedo‐Whitehouse et al., 2006; Hawley, Sydenstricker, Kollias, & Dhondt, 2005), and survival (Keller, Grant, Grant, & Petren, 2002; Olano‐Marin, Mueller, & Kempenaers, 2011a). The positive association between individual genetic diversity and fitness‐related traits arises when homozygotes are less fit than heterozygotes due to either heterozygote advantage (i.e., overdominance) or recessivity of deleterious or partly deleterious alleles (Charlesworth & Charlesworth, 1987; Keller & Waller, 2002). The study of this relationship in wild populations has traditionally been problematic due to the difficulty of obtaining well‐resolved pedigrees to estimate individual co‐ancestry (Pemberton, 2004). An alternative approach, consisting of measuring genetic diversity at a set of marker loci, has thus become popular (reviewed in Chapman, Nakagawa, Coltman, Slate, & Sheldon, 2009). Consequently, in most studies, individual genetic diversity is assessed using microsatellite markers, which are only expected to reflect genomewide heterozygosity if different processes, fundamentally inbreeding, genetic drift, genetic admixture, and bottlenecks, contribute to the generation of identity disequilibrium (ID) (Balloux, Amos, & Coulson, 2004; Szulkin, Bierne, & David, 2010). Although ID is considered to be the fundamental cause of heterozygosity–fitness correlations (HFC) (“general effect hypothesis”; David, 1998), it has been suggested that HFC may also result from functional overdominance at the scored loci per se (“direct effect hypothesis”; David, 1998; Li, Korol, Fahima, & Nevo, 2004) or as a consequence of some markers being linked to genes under selection (“local effect hypothesis”; García‐Navas, Cáliz‐Campal, Ferrer, Sanz, & Ortego, 2014; Hansson & Westerberg, 2002; Slate et al., 2004). Although a considerable number of studies have analyzed the association between different components of fitness and marker‐based estimates of heterozygosity, the relative importance of the above‐described hypotheses to explain observed HFC is still controversial and a matter of ongoing debate (Chapman et al., 2009; Miller & Coltman, 2014; Szulkin et al., 2010).

The extent of inbreeding depression and the magnitude of HFC have been suggested to depend on the specific environmental context in which they are assayed (Armbruster & Reed, 2005; Fox & Reed, 2011; Marr, Arcese, Hochachka, Reid, & Keller, 2006; Miller, 1994). Different studies have found that inbreeding depression and HFC only arise or manifest more strongly when resources are limited, for example, food supply (Lesbarrères, Primmer, Laurila, & Merilä, 2005), or under stressful conditions imposed by different biotic (parasites: Coltman, Pilkington, Smith, & Pemberton, 1999; Voegeli, Saladin, Wegmann, & Richner, 2012; competitors: Cheptou, Imbert, Lepart, & Escarre, 2000) and abiotic factors (thermal stress: Scott & Koehn, 1990; adverse weather conditions: Forcada & Hoffman, 2014; Marr et al., 2006). Thus, the environmental context (i.e., the “stressfulness” of the environment) under which HFC are analyzed can have profound effects on the inferred consequences of reduced levels of genetic diversity for a given population. In this sense, the high spatiotemporal variability in the conditions experienced by individuals (e.g., weather, parasitism pressure, and competition) could explain the apparently contradictory results found in different HFC studies on wild populations (Armbruster & Reed, 2005; Chapman et al., 2009; Coltman & Slate, 2003; Fox & Reed, 2011). The study of genetic diversity–environment interactions also has important implications for understanding the potential of populations to cope with different sources of stress and predict their future demographic and evolutionary dynamics in response to climate change and habitat fragmentation (Forcada & Hoffman, 2014; Liao & Reed, 2009; Reed, Briscoe, & Frankham, 2002; Reed, Nicholas, & Stratton, 2007). However, studies on HFC performed in natural populations have rarely considered the impact of environmental heterogeneity, and evidence on this respect generally comes from experimental or laboratory studies (but see Coltman et al., 1999; Forcada & Hoffman, 2014; Marr et al., 2006).

**Figure 1:**
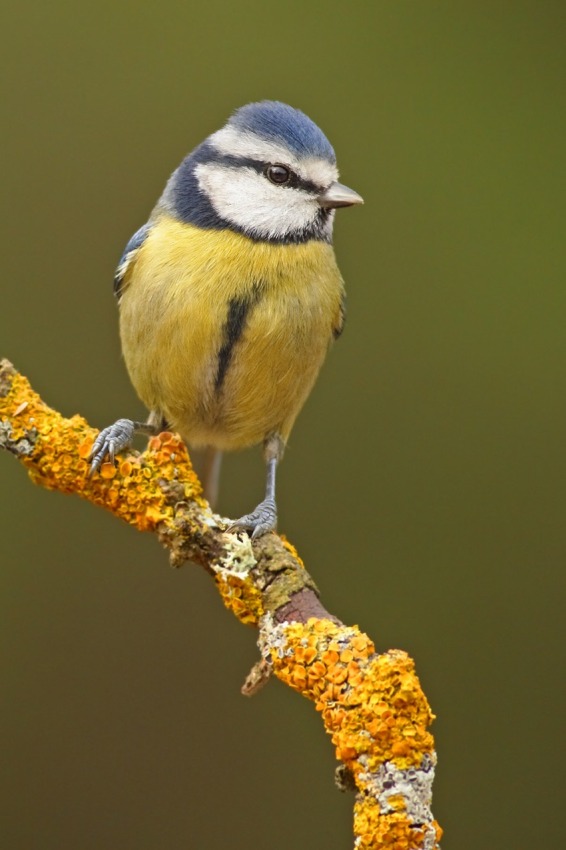
Blue tit (*Cyanistes caeruleus*), the study organism. Photograph by Juan Caballero López

In the present study, we combine extensive molecular and capture–mark–recapture data to analyze the relationship between heterozygosity and probability of interannual adult local recruitment in a short‐lived passerine with limited dispersal, the blue tit (*Cyanistes caeruleus*) (Figure 1). Previous studies carried out on this species found significant heterozygosity–fitness correlations considering different life‐history traits (reproductive performance: Foerster, Delhey, Johnsen, Lifjeld, & Kempenaers, 2003; García‐Navas, Ortego, & Sanz, 2009; Olano‐Marin, Mueller, & Kempenaers, 2011b; survival probability: Olano‐Marin, Mueller, & Kempenaers, 2011a; and parasite resistance: Ferrer, García‐Navas, Sanz, & Ortego, 2014). Consanguineous matings have been reported in these populations (Ferrer et al., 2014; García‐Navas et al., 2009), which may increase variance in inbreeding and the chance of detecting HFC due to extensive ID across the genome (Balloux et al., 2004; Szulkin et al., 2010). In addition, we examine the relationship between environmental variability and the strength of HFC. As with other small passerines, the blue tit has a high metabolic rate and a limited capacity to deposit long‐term body reserves (McNab, 2002), factors that are likely to increase its susceptibility to environmental stress imposed by food shortage or severe weather conditions (Marr et al., 2006). For these reasons, our study system is particularly amenable to study HFC and their interactions with environment.

Specifically, we monitored interannual local recruitment over six consecutive years (2008–2013) and genotyped all adult blue tits across 26 microsatellite markers to estimate individual genetic diversity. Further, these microsatellite markers were classified as putatively neutral (14 loci) or functional (12 loci) by considering whether the genomic region where they are located is transcribed to RNA (sensu Olano‐Marin, Mueller, & Kempenaers, 2011a; Olano‐Marin, Mueller, & Kempenaers, 2011b), which allowed us to test differences in HFC and genetic diversity–environment interactions between these subsets of loci (see Methods for more details on the employed loci and their classification). Neutral markers can produce HFC either by general effects or by local effects if they happen to be linked to functional loci, whereas direct effects and strong local effects are only likely to be caused by functional markers (Ferrer et al., 2014; Olano‐Marin, Mueller, & Kempenaers, 2011a; Olano‐Marin, Mueller, & Kempenaers, 2011b). First, we analyzed (1) the relationship between probability of interannual local recruitment and individual genetic diversity and tested whether such associations (2) varied between putatively functional and neutral loci and (3) were better explained by a genomewide effect (“general effect hypothesis”) or strong linkage disequilibrium between the employed markers and genes under selection (“local effect hypothesis”). Second, (4) we studied whether environmental stress imposed by physiologically suboptimal temperatures and rainfall influence the magnitude of HFC (e.g., Forcada & Hoffman, 2014). Finally, (5) we analyzed whether selection on heterozygosity has resulted in a change in population genetic diversity over time (e.g., Bensch et al., 2006; Forcada & Hoffman, 2014; Kaeuffer, Coltman, Chapuis, Pontier, & Réale, 2007).

## 2 Materials and Methods

### 2.1 Study system

During seven consecutive years (2008–2014), we monitored a blue tit population established in two nearby plots (Gil García: 39°22′N 4°07′W; Valdeyernos: 39°26′N 4°05′W) located in Quintos de Mora (Montes de Toledo, central Spain). Both study plots consist of a Pyrenean oak (*Quercus pyrenaica*) forest (~22 ha) containing 100 nestboxes (see Figure 1 in Ferrer et al., 2016 for a map of the study area). The study plots are separated by 7 km, and their respective populations show a low degree of genetic differentiation (*F*_ST_ = 0.005; Ferrer et al., 2016). The climate is meso‐Mediterranean with mean temperatures ranging from 24–26°C in July to 4–6°C in January and 300–600 mm of rainfall concentrated in spring and autumn. Regular frosts take place at night during the winter, but temperatures very rarely fall below −5°C, and snowfalls are anecdotal (Tornero, 2003). Nestboxes were checked regularly from early April to late June in order to determine general breeding parameters, including laying date, clutch size, hatching date, hatching success, and fledging success. Adults were captured once during the breeding season exclusively by means of spring traps when feeding nestlings 8–9 days of age. Birds were weighed using an electronic portable balance (accuracy ±0.1 g), and their wing length (accuracy ±1 mm) and tarsus length (accuracy ±0.1 mm) were measured using a top ruler and a digital caliper, respectively. Blood samples (≤25 μl) were obtained by puncturing the brachial vein and stored on FTA reagent‐loaded cards (Whatman Bioscience, Florham Park, NJ, USA) or in Eppendorf tubes with 96% ethanol until needed for genetic analyses. Birds were aged according to Svensson (1992) as juveniles (yearlings) or adults (second‐year or older birds). We knew the exact age of 73% of individuals that were ringed as fledglings or juveniles. For all other individuals, we considered that they were captured in their second year if they presented adult plumage (e.g., Ortego et al., 2009). All adults and nestlings were individually marked with aluminum rings for further identification. Individuals ringed as nestlings that recruited as breeders in the same population were classified as locals, whereas all other individuals (i.e., adult birds born in a different population or that were unringed the first time they were captured) were considered to be immigrants.

### 2.2 Interannual local recruitment estimates

Interannual local recruitment was assessed through capture–mark–recapture of breeding individuals across all the study years (capture effort = 82.72%) under the assumption that individuals not returning to the study area in the subsequent year had died (local recruitment = local recruitment the following year). Individuals recaptured the following breeding season in any nestbox plot from the study area (see Figure 1 in Ferrer et al., 2016) were coded as “1”, whereas those that were not recovered in the next year were coded as “0” (e.g., Olano‐Marin, Mueller, & Kempenaers, 2011a). Our capture effort (i.e., the proportion of individuals captured among all those breeding in our study populations) was high (82.72%), and as a result, the proportion of birds classified as “nonrecruited” but returning as breeders in subsequent years (i.e., individuals that actually survived but were not captured in year *t *+* *1) was low (13.1%). We adopted this approach in order to (1) avoid that our models become less accurate over years and (2) keep the errors around our estimates of local recruitment comparable across years. These aspects are paramount for this study, as our main aim was to compare the effects of environmental harshness on the strength of HFC across years. Around 20% of nestboxes remain unoccupied every year, so we are confident that nest site shortage is not promoting high rates of breeding dispersal beyond our study area, which may affect our estimates of interannual local recruitment. Accordingly, most individuals of our populations are highly philopatric, and we have only recorded two instances of breeding dispersal between Gil García and Valdeyernos since the beginning of our study. It should be also noted that our study plots are fairly ecologically isolated as they constitute deciduous forest remnants in a matrix of habitat sustaining very low breeding densities of forest passerines due to either the presence of unsuitable habitat (Mediterranean scrublands and croplands) or the shortage of natural cavities for breeding in the scattered patches of immature forests present in this area (Ferrer et al., 2016; García‐Navas, Ferrer, Bueno‐Enciso et al., 2014).

### 2.3 Microsatellite genotyping

We genotyped a total of 388 blue tits using a panel of 26 polymorphic microsatellite markers distributed across 10 chromosomes (Table S1). Following the approach detailed in the study of Olano‐Marin, Mueller, and Kempenaers (2011a), Olano‐Marin, Mueller, and Kempenaers (2011b), we classified our markers as presumably functional or neutral by considering whether the genomic region where they are located is transcribed to RNA (Table S1) (Ferrer et al., 2014; Olano‐Marin, Mueller, & Kempenaers, 2011a; Olano‐Marin, Mueller, & Kempenaers, 2011b). More precisely, loci that were designed based on or showed homology to zebra finch (*Taeniopygia guttata*). Expressed sequence tags (ESTs) were considered to be putatively functional, whereas markers designed using traditional cloning methods and with no homology to avian ESTs were considered to be neutral (for more details on loci development, see Olano‐Marin et al., 2010). It must be emphasized two important questions regarding this approach. On the one hand, we refer to presumably functional microsatellite loci as those located within genomic regions that are transcribed to RNA, but we have no information on whether they are functional themselves (sensu Li et al., 2004). On the other hand, we did not aim to employ a “candidate gene approach” (e.g., to link fitness with specific genes/alleles with known function) or target microsatellite markers linked to a specific gene, but just to compare our two subsets of loci classified according with the above‐described criteria (e.g., Ferrer, García‐Navas, Bueno‐Enciso, Sanz, & Ortego, 2015; Ferrer et al., 2014; Olano‐Marin, Mueller, & Kempenaers, 2011a; Olano‐Marin, Mueller, & Kempenaers, 2011b). DNA extraction, microsatellite amplification, and genotyping and tests for deviations from Hardy–Weinberg equilibrium (HWE) and linkage disequilibrium (LD) between each pair of loci were performed as described in Ferrer et al. (2014).

### 2.4 Heterozygosity estimates and identity disequilibrium

We used homozygosity by locus (HL) to estimate individual genetic diversity, an index that improves heterozygosity estimates in open populations by weighting the contribution of each locus to the homozygosity value depending on their allelic variability (Aparicio, Ortego, & Cordero, 2006). We calculated HL for all typed markers (HL_Total_) and separately for the subsets of neutral (HL_Neutral_) and functional loci (HL_Functional_). HL values were calculated using cernicalin, an excel spreadsheet available online at: https://sites.google.com/site/joaquinortegolozano/software-1. We used the inverse of HL (i.e., 1‐HL) as an estimate of individual heterozygosity in subsequent analyses. To analyze the presence of identity disequilibrium in our population and determine whether heterozygosity measured at our set of microsatellite loci is representative of genomewide inbreeding, we calculated the parameter *g* ^2^ and tested whether it differed significantly from zero using 1,000 iterations in the software rmes (David, Pujol, Viard, Castella, & Goudet, 2007).

### 2.5 Heterozygosity–fitness correlations: multilocus effects

We used an information‐theoretic model selection approach to analyze the association between probability of interannual local recruitment and heterozygosity (Burnham & Anderson, 2002). For each year, we constructed two separate generalized linear mixed models (GLMMs, binomial error and logit link function), one including as predictor variable individual heterozygosity (i.e., 1‐HL) calculated for all loci (HL_Total_) and another including as predictor variables heterozygosity estimated for the subsets of neutral (HL_Neutral_) and functional (HL_Functional_) markers (e.g., Ferrer et al., 2014). Study plot, sex, and the origin of individuals (local/immigrant) were included as fixed factors in all the models. Moreover, we included as covariates fledging success (i.e., number of fledged young), age of individuals (since birth, estimated as indicated in Methods) and body condition. Body condition was estimated as the residuals of a multiple regression of body mass on laying date and wing and tarsus length in order to correct body mass for the timing of breeding and structural body size, respectively. We also included mating pair identity as random effect in all the models to take into account that individuals sharing the same territory (i.e., mating pairs) may be affected by similar environmental factors influencing their probability of local recruitment (e.g., the presence of the same predators in the territory, equal food availability, etc.). AICc values for each model were rescaled (ΔAICc) calculating the difference between the AICc value of each model and the minimum AICc obtained among all competing models (i.e., the best model has ΔAICc = 0). Models with ΔAICc ≤ 2 were considered equivalent (Burnham & Anderson, 2002; e.g., Ferrer et al., 2015). Model selection and averaging were performed using the R packages lme4 (Bates, Maechler, Bolker, & Walker, 2015) and AIccmodavg (Mazerolle, 2014; R Core Team, 2012).

### 2.6 Heterozygosity–fitness correlations: single‐locus effects

First, we examined whether multilocus heterozygosity (MLH) explained more variance than single‐locus heterozygosity (SLH) following the approach described in the study of Szulkin et al. (2010). We performed *F*‐ratio tests to compare models including MLH with those in which we replaced MLH with “normalized” SLH at all markers for each year (David, 1997; Szulkin et al., 2010). Second, we analyzed the effect of single‐locus heterozygosity (SLH) by fitting one GLMM per locus and year and applied sequential Bonferroni corrections (Rice, 1989) to account for the remarkably high number of statistical tests performed (26 markers × 6 years = 156 tests). Bonferroni corrections were not used for other statistical analyses due to the expected very small effect sizes (i.e., low statistical power) in HFC studies on natural populations (Balloux et al., 2004; Chapman et al., 2009) and the unacceptably high probability of making Type II errors if such corrections are applied (see Nakagawa, 2004). Effect size was calculated for each locus as the partial correlation coefficient obtained from its respective model (Nakagawa & Cuthill, 2007). Finally, we used a general linear model (GLM) to analyze whether absolute effect sizes of single‐locus heterozygosities were associated with marker variability (allelic richness and observed heterozygosity, included as covariates) and differed between neutral and putatively functional loci (marker category was included as a fixed factor) (e.g., Ferrer et al., 2014; Olano‐Marin, Mueller, & Kempenaers, 2011a; Olano‐Marin, Mueller, & Kempenaers, 2011b).

### 2.7 Environmental data

Meteorological data used to determine the local environmental conditions experienced by individuals outside the breeding season, that is, from the end of reproduction (July 1) until the beginning of the next breeding season (March 31) were obtained from the meteorological station located in Quintos de Mora (39° 25′N, 4° 04′W). We calculated three parameters as potential indicators of environmental harshness and that may influence the survival prospects and/or the individual physiological state in our study species. First, we calculated the number of days with temperature ≥35°C or ≤15°C. These temperatures represent the upper and lower critical limits of the thermal neutral zone (TNZ; i.e., the temperature tolerance range of an endotherm organism) for blue tits and other small passerines beyond which energy expenditure and oxygen consumption increase (e.g., Gavrilow & Dolnik, 1985; Thomas, Blondel, & Perret, 2001). Second, we calculated the number of days with temperatures below 0°C (freezing‐degree days, FDD), because frosts are likely to considerably increase energy expenditure for thermoregulation, particularly in Mediterranean small passerines adapted to mild climates (Broggi et al., 2007). Third, we calculated the accumulated precipitation (in millimeters, mm). Rainfall can dramatically increase energy demands for thermoregulation and energy expenditure for flight (through reduced maneuverability as feathers become water‐logged) (Wilson, Cooper, & Gessaman, 2004). In addition, rainfall can reduce food availability and birds’ capacity to acquire this resource, particularly in insectivorous species because arthropods are less active during adverse weather (e.g., Arlettaz, Schaad, Reichlin, & Schaub, 2010; Avery & Krebs, 1984), which can lead to a reduction in body condition and increase the likelihood of starvation and mortality (Öberg et al., 2015). Our three estimates of environmental harshness were not significantly intercorrelated (all *p*s > .33).

### 2.8 Selection analyses

The strength of the association between heterozygosity and fitness is expected to depend on factors such as the intensity of selection and the level of ID in the population (Armbruster & Reed, 2005; Marr et al., 2006; Fox et al. 2011). For each year, we estimated the directional selection differentials (*S*) (Falconer & Mackay, 1996; Matsumura, Arlinghaus, & Dieckmann, 2012) for heterozygosity in relation to probability of interannual local recruitment, a component of fitness. Selection differentials were calculated for each year as the covariance of individual heterozygosity and standardized probability of local recruitment (i.e., *S *= cov[ω,heterozygosity]). Selection differentials were estimated for all markers (*S*_Total_) and the subsets of neutral (*S*_Neutral_) and functional (*S*_Functional_) loci separately (positive higher values = greater selection by heterozygosity). We then analyzed the association between *S* and the three cues indicative of environmental harshness described in the previous section (see Section 2.7) using simple linear regressions. Thereby, we tested whether environmental severity intensifies the strength of selection on heterozygosity. Finally, we used simple linear regressions to analyze whether selection on heterozygosity has resulted in a change in annual mean heterozygosity values over time (e.g., Bensch et al., 2006; Forcada & Hoffman, 2014; Kaeuffer et al., 2007). These analyses must be interpreted with caution and always considering that selection differentials were calculated on the basis of a single component of fitness that may not necessarily reflect true fitness and actual selection on heterozygosity (e.g., Bensch et al., 2006).

## 3 Results

### 3.1 Patterns of interannual adult local recruitment

Rates of interannual local recruitment ranged between 18.48% and 50% (2008–2009: 50%; 2009–2010: 38%; 2010–2011: 44%; 2011–2012: 37%; 2012–2013: 18%; 2013–2014: 28%). The lowest rate of interannual local recruitment was for 2012–2013, corresponding with the period with the highest accumulated precipitation and one with the highest number of freezing‐degree days. However, when we analyzed the relationship between annual rates of local recruitment and the variables used as proxies for environmental harshness, we only found a marginally significant trend with FDD (TNZ: *r = *.106, *p *=* *.842; FDD: *r *=* *−.774, *p *=* *.070; accumulated precipitation: *r *=* *−.394, *p *=* *.439).

### 3.2 Basic genetic statistics

Observed heterozygosity at each locus ranged from 0.48 to 0.94, with 3–26 alleles per locus (Table S1). Neutral loci tended to have higher allelic richness (*A*_R_) and observed heterozygosity (*H*_O_) than putatively functional loci, but these differences were not statistically significant (one‐way ANOVAs, *A*_R_: *F*_1,24_ = 3.60, *p *=* *.069; *H*_O_: *F*_1,24_ = 2.77, *p *=* *.108). No loci consistently deviated from HWE or exhibited LD across all years, and therefore, all markers were used in subsequent analyses (see also Ferrer et al., 2014).

### 3.3 Identity disequilibrium

The *g* ^2^ estimator of identity disequilibrium calculated for all markers was positive in all study years and differed significantly from zero in 2008, 2009, and 2012 (Table S2). When the subset of neutral loci was analyzed separately, *g* ^2^ values were also always positive and differed significantly from zero in 2009 and 2010 and were marginally nonsignificant in 2011 (*p *=* *.087) and 2012 (*p *=* *.077) (Table S2). For the subset of functional markers, *g* ^2^ value was only positive in 2011, 2012, and 2013 and only differed significantly from zero in 2012 (Table S2). These results suggest that marker heterozygosity is representative of genomewide heterozygosity in most years in our study on blue tit population (Szulkin et al., 2010).

### 3.4 Heterozygosity–fitness correlations: multilocus effects

**Figure 2:**
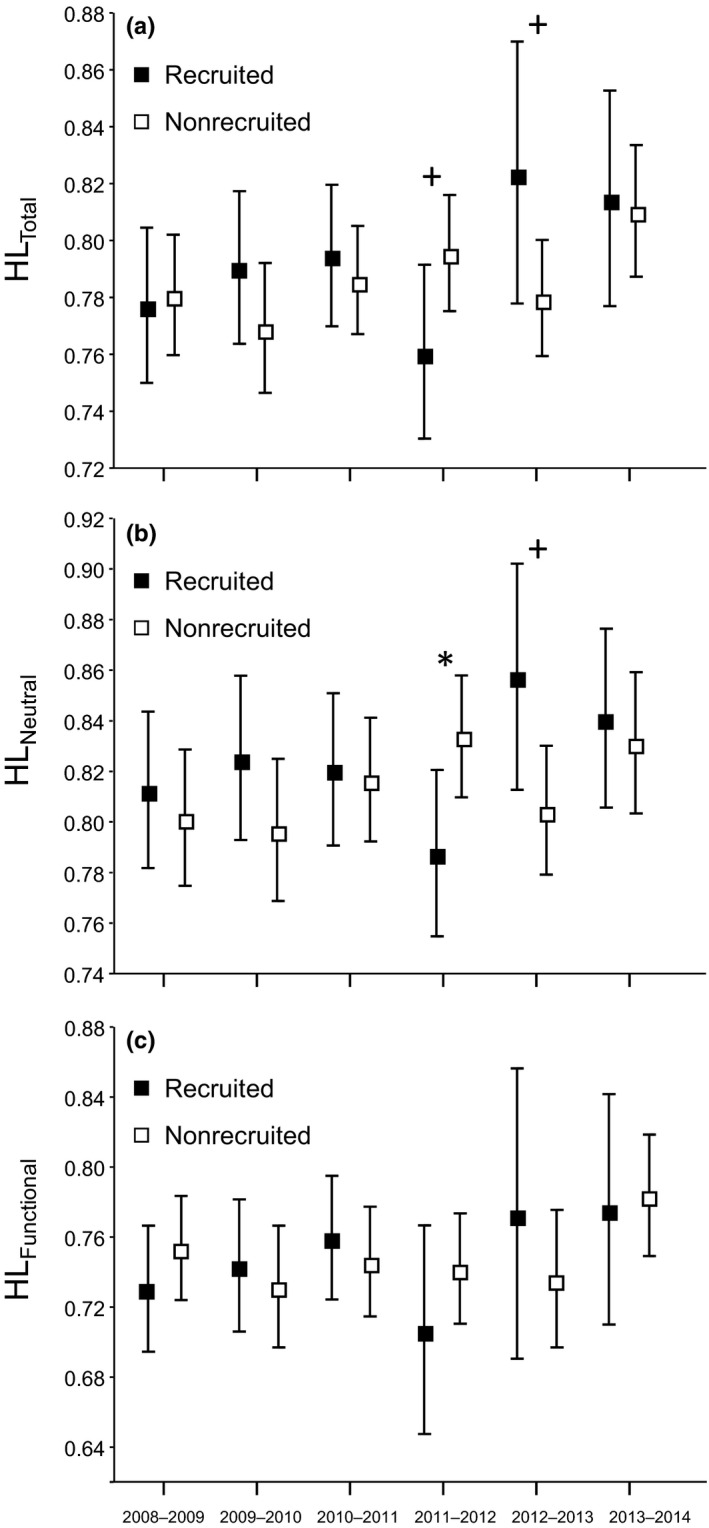
Mean (±SE) heterozygosity of individuals that recruited and nonrecruited into the local populations between two consecutive years. Panels show heterozygosity estimated at (a) all markers (HL_Total_) and the subsets of (b) neutral (HL_Neutral_) and (c) putatively functional loci (HL_Functional_). Significance of univariate logistic regressions (only including heterozygosity as covariate) for each year and subset of markers is indicated (*p <* *.05; ^+^ *p *<* *.1)

Probability of local recruitment was significantly associated with individual heterozygosity estimated at all loci in 2011 and 2012 (Tables 1 and S3) and with heterozygosity estimated at the subset of neutral markers in 2011 (Tables 2 and S4). Yet, heterozygosity estimated at the subset of functional loci had no significant effect on probability of local recruitment in any year (Table 2). The direction of the association differed between years; probability of local recruitment decreased with individual heterozygosity in 2011, and the opposite pattern was found the next year (Tables 1 and 2). Regarding nongenetic terms, we found that in some models the probability of local recruitment was positively associated with body condition, males were more likely to recruit than females, and the likelihood of interannual local recruitment was higher in Gil García than in Valdeyernos (Tables 1 and 2). However, we did not find any significant association between probability of interannual local recruitment and fledgling success or the immigrant/local status of individuals (Tables 1 and 2). The interaction between heterozygosity and locality had no significant effect in any year (i.e., CIs spanned zero in all cases), indicating that the observed HFC are genuine and have not resulted from population stratification (Slate & Pemberton, 2006; Slate et al., 2004). Neither was the interaction between heterozygosity and sex significant for any study year, indicating that the association between probability of local recruitment and individual genetic diversity did not differ between males and females. The random effect (pair identity) had a little impact on our models, and analyses ran excluding it yielded qualitatively similar results in terms of both model selection and the significance of the variables included (data not shown) (Bolker et al., 2009). We performed an analysis with data from all years collectively. This analysis did not show any significant effect of heterozygosity on the probability of local recruitment (*p *>* *.3), probably due to the presence of opposite trends in different years (Tables 1 and 2; Figure 2). When the data were analyzed using univariate logistic regressions that only included heterozygosity as covariate, the observed associations between individual genetic diversity and probability of local recruitment were qualitatively similar to those obtained in multivariate analyses (Figure 2). In analyses only including individual genetic diversity as covariate, the interaction between sex and heterozygosity was not significant in any year (all *p*s > .1). Overall, this indicates that the results from multivariate analyses are not much influenced by interactions among independent variables.

**Table 1:** GLMMs (binomial error and logit link function) for probability of interannual local recruitment (i.e., local recruitment from year *t* to year *t *+* *1) in relation to heterozygosity estimated at all loci (HL_Total_) and different nongenetic terms (locality, sex, age, body condition, fledging success, and local/immigrant status)

	Estimate ± USE	Σ ωi	Lower95% CI	Upper95% CI
(a) Local recruitment from 2008 to 2009 breeding season				
Sex	1.47 ± 0.70	0.17	**0.10**	**2.84**
Fledging success	0.32 ± 0.20	0.17	−0.06	0.71
Local/Immigrant	1.48 ± 0.86	0.17	−0.20	3.16
(b) Local recruitment from 2009 to 2010 breeding season				
Locality	0.93 ± 0.42	0.26	**0.11**	**1.76**
Body condition	1.04 ± 0.44	0.26	**0.18**	**1.91**
HL_Total_	3.54 ± 2.20	0.24	−0.78	7.86
Local/Immigrant	0.42 ± 0.42	0.11	−0.42	1.25
Fledging success	−0.07 ± 0.09	0.05	−0.24	0.09
(c) Local recruitment from 2010 to 2011 breeding season				
Body condition	−0.26 ± 0.41	0.36	−1.07	0.55
Local/Immigrant	0.65 ± 0.51	0.12	−0.35	1.65
Locality	0.61 ± 0.53	0.11	−0.43	1.63
Sex	0.25 ± 0.48	0.04	−0.70	1.19
Age	−0.23 ± 0.30	0.04	−0.82	0.36
(d) Local recruitment from 2011 to 2012 breeding season				
HL_Total_	−5.79 ± 2.49	0.28	−**10.67**	−**0.90**
Body condition	−0.56 ± 0.40	0.28	−1.33	0.22
Locality	0.68 ± 0.51	0.08	−0.32	1.68
Local/Immigrant	0.37 ± 0.47	0.05	−0.55	1.29
Age	0.08 ± 0.23	0.04	−0.36	0.53
(e) Local recruitment from 2012 to 2013 breeding season				
HL_Total_	10.55 ± 4.84	0.31	**1.07**	**20.03**
Locality	1.89 ± 0.91	0.31	**0.10**	**3.68**
Age	−0.64 ± 0.43	0.31	−1.48	0.19
Body condition	−0.23 ± 0.72	0.31	−1.64	1.18
Sex	0.61 ± 0.79	0.10	−0.95	2.16
(f) Local recruitment from 2013 to 2014 breeding season				
Body condition	0.49 ± 0.54	0.24	−0.56	1.54
Fledging success	0.11 ± 0.19	0.06	−0.25	0.48
Age	0.06 ± 0.52	0.05	−0.96	1.09

Mating pair identity was included as random effect in all the models. We performed model averaging of the best ranked equivalent models (ΔAICc ≤ 2) to obtain parameter estimates and unconditional standard errors (USE) (see Table S3). Variables are sorted according to their relative importance based on the sum of Akaike weights (Σ ωi) of those models with ΔAICc ≤ 2 in which the variable was present. Bold type indicates significant variables, that is, variables for which their unconditional 95% confidence interval (CI) did not cross zero.

**Table 2:** GLMMs (binomial error and logit link function) for probability of interannual local recruitment (i.e., local recruitment from year *t* to year *t *+* *1) in relation to heterozygosity estimated at the subset of neutral (HL_Neutral_) and putatively functional (HL_Functional_) loci and different nongenetic terms (locality, sex, age, body condition, fledging success, and local/immigrant status)

	Estimate ± USE	Σ ωi	Lower95% CI	Upper95% CI
(a) Local recruitment from 2008 to 2009 breeding season				
Sex	1.49 ± 0.70	0.15	**0.12**	**2.86**
Fledging success	0.32 ± 0.19	0.15	−0.66	0.69
Local/Immigrant	1.49 ± 0.86	0.15	−0.18	3.17
(b) Local recruitment from 2009 to 2010 breeding season				
Locality	0.94 ± 0.42	0.39	**0.11**	**1.76**
Body condition	1.05 ± 0.44	0.39	**0.18**	**1.92**
HL_Neutral_	2.77 ± 1.80	0.21	−0.77	6.30
Local/Immigrant	0.44 ± 0.43	0.10	−0.40	1.28
HL_Functional_	1.03 ± 1.51	0.07	−1.94	3.99
Fledging success	−0.08 ± 0.09	0.07	−0.25	0.09
(c) Local recruitment from 2010 to 2011 breeding season				
Body condition	−0.29 ± 0.28	0.33	−0.84	0.26
Local/Immigrant	0.65 ± 0.47	0.13	−0.27	1.57
Locality	0.62 ± 0.50	0.08	−0.36	1.60
Age	−0.22 ± 0.29	0.06	−0.79	0.35
HL_Functional_	1.72 ± 2.00	0.03	−2.21	5.64
Sex	0.29 ± 0.44	0.03	−0.56	1.14
(d) Local recruitment from 2011 to 2012 breeding season				
HL_Neutral_	−5.14 ± 2.47	0.22	−**9.99**	−**0.30**
HL_Functional_	−1.73 ± 1.83	0.22	−5.31	1.83
Body condition	−0.51 ± 0.46	0.15	−1.41	0.40
Locality	0.90 ± 0.57	0.15	−0.21	2.01
Age	0.20 ± 0.23	0.05	−0.26	0.66
(e) Local recruitment from 2012 to 2013 breeding season				
Locality	2.06 ± 0.94	0.42	**0.21**	**3.91**
HL_Neutral_	7.10 ± 3.68	0.42	−0.11	14.31
HL_Functional_	3.75 ± 2.63	0.42	−1.40	8.89
Age	−0.64 ± 0.45	0.24	−1.51	0.23
Body condition	−0.09 ± 0.75	0.18	−1.57	1.38
Local/Immigrant	−1.37 ± 0.91	0.11	−3.16	0.42
Sex	0.85 ± 0.84	0.07	−0.79	2.49
(f) Local recruitment from 2013 to 2014 breeding season				
HL_Neutral_	92.35 ± 73.94	0.15	−52.56	237.26
HL_Functional_	12.77 ± 32.24	0.15	−50.43	75.96
Local/Immigrant	8.37 ± 9.94	0.15	−11.11	27.86

Mating pair identity was included as random effect in all the models. We performed model averaging of the best ranked equivalent models (ΔAICc ≤ 2) to obtain parameter estimates and unconditional standard errors (USE) (see Table S4). Variables are sorted according to their relative importance based on the sum of Akaike weights (Σ ωi) of those models with ΔAICc ≤ 2 in which the variable was present. Bold type indicates significant variables, that is, variables for which their unconditional 95% confidence interval (CI) did not cross zero.

### 3.5 Heterozygosity–fitness correlations: single‐locus effects

We did not find significant differences in the variance explained by the models including multilocus heterozygosity (MLH) compared to the models including single‐locus heterozygosity (SLH) in any year or considering any subset of loci (all *p*s > .1). Furthermore, no single‐locus effect was significant after sequential Bonferroni correction (Table S5). Absolute effect size of SLH was not correlated with marker genetic diversity (estimated as *H*_O_ and *A*_R_; Table S1) and did not differ between the subsets of neutral and functional loci in any year (all *p*s* *> .05).

### 3.6 Selection analyses

**Figure 3:**
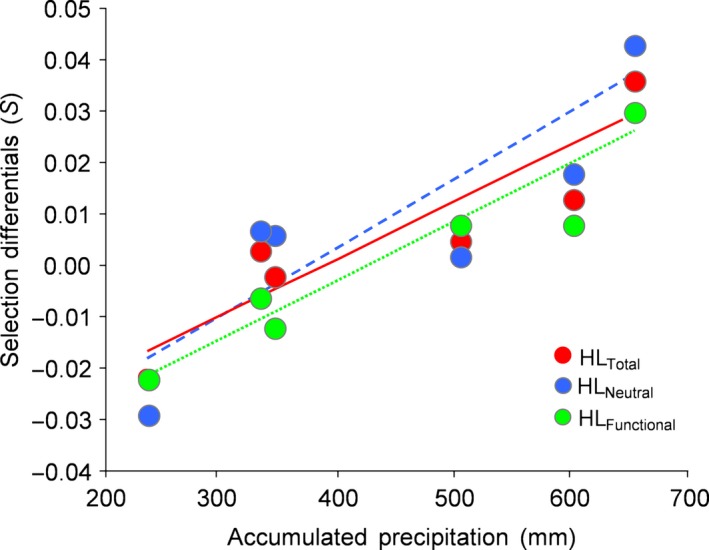
Relationship between annual accumulated precipitation in each year and selection differentials (*S*) for heterozygosity estimated at all markers (HL_Total_) and the subsets of neutral (HL_Neutral_) and putatively functional loci (HL_Functional_). Regression lines are shown

Selection differentials (*S*) estimated at all loci were positive in all years except for 2008 and 2011, for neutral loci were positive in all years except 2011 and for putatively functional loci were negative in 2008, 2011, and 2013 (Table S2). *S* estimated at neutral and functional loci were significantly correlated (*r *=* *.897, *p *=* *.015), suggesting that selection on heterozygosity acts in a similar way in both sets of markers. When we analyzed the association between *S* and the variables used as proxies for environmental harshness, we found a positive association with annual accumulative precipitation for all the subsets of markers (Table 3; Figure 3). This indicates that selection on heterozygosity was higher in years with harder climatic conditions, that is, wetter years. However, *S* was not associated with the number of days that reached temperatures below 0°C or beyond the limits of the thermal neutral zone (Table 3).

**Table 3:** Linear regressions (*r* values) between the three studied parameters associated with environmental harshness for each year (TNZ days: number of days with temperature ≥35°C or ≤15°C; FDD: freezing‐degree days, calculated as the number of days with temperature <0°C; accumulated precipitation) and selection differentials (*S*) for heterozygosity estimated at all markers (HL_Total_) and the subsets of neutral (HL_Neutral_) and putatively functional loci (HL_Functional_) for each study year

	TNZ days	FDD	Accumulated precipitation
HL_Total_	0.294 (*p *=* *.572)	0.050 (*p *=* *.926)	0.911 (*p *=* *.011)
HL_Neutral_	0.321 (*p *=* *.535)	0.043 (*p *=* *.935)	0.862 (*p *=* *.027)
HL_Functional_	0.232 (*p *=* *.658)	0.104 (*p *=* *.845)	0.946 (*p *=* *.004)

*p*‐Values are indicated in parentheses.

**Figure 4:**
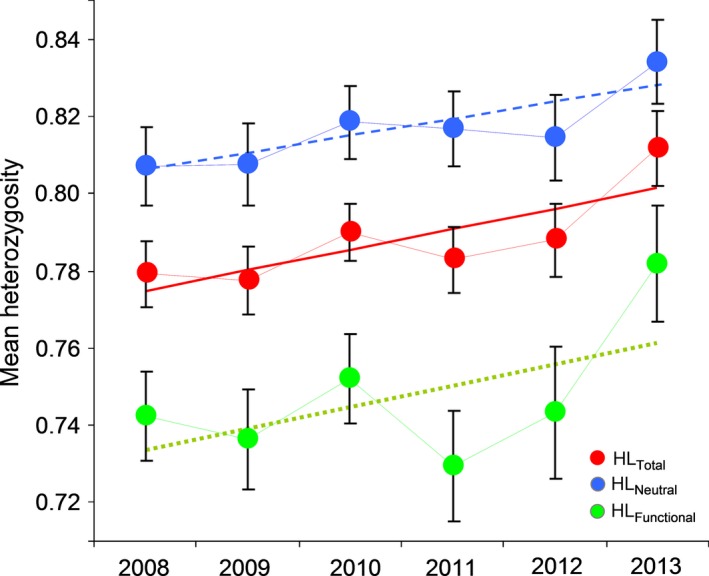
Temporal change in mean (±SE) heterozygosity estimated at all markers (HL_Total_) and the subsets of neutral (HL_Neutral_) and putatively functional loci (HL_Functional_) over the study period. Trend lines are shown

Annual mean heterozygosity estimated at neutral and functional loci were significantly correlated (*r *=* *.844, *p *=* *.034). Annual mean heterozygosity values estimated at the subset of neutral loci increased significantly over the course of the study period (HL_Neutral_: *r *=* *.831, *p *=* *.040) and similar but nonsignificant trends were obtained for annual mean heterozygosity estimated at all loci (HL_Total_: *r *=* *.799, *p *=* *.056) and the subset of functional markers (HL_Functional_: *r *=* *.566, *p *=* *.240) (Figure 4).

## 4 Discussion

We found significant relationships between individual genetic diversity estimated using different subsets of markers and adult local recruitment probability in our study system. However, the direction and magnitude of HFC differed among years and they were only significantly explained by multilocus heterozygosity calculated at all markers or the panel of neutral markers, but not by genetic diversity estimated at the subset of putatively functional loci. We also found a highly significant positive relationship between selection differentials (*S*) for heterozygosity and annual accumulated precipitation, which indicates that interannual differences in HFC may be explained by the different environmental conditions to which individuals are exposed each year (Armbruster & Reed, 2005; Fox & Reed, 2011; Marr et al., 2006). Hence, our results reinforce the expectation of stronger selection under adverse environmental conditions in wild populations (Endler, 1986). We also found that heterozygosity increased over time, suggesting a micro‐evolutionary response to selection over the course of the study period (Bensch et al., 2006; Forcada & Hoffman, 2014; Kaeuffer et al., 2007).

### 4.1 Heterozygosity and probability of local recruitment

We found different effects of multilocus heterozygosity on probability of local recruitment depending on the year of study. Probability of local recruitment from 2012 to 2013 breeding season was positively associated with individual genetic diversity. Individuals that bred in 2012 had to cope with a severe winter (high rainfall and incidence of FDD above the average; Table S2), which may have resulted in stronger natural selection against homozygous individuals (Armbruster & Reed, 2005; Fox & Reed, 2011; Keller, Arcese, Smith, Hochachka, & Stearns, 1994). On the contrary, we found a counterintuitive negative association between heterozygosity and probability of local recruitment from 2011 to 2012 breeding season. Individuals that bred in 2011 experienced more benign environmental conditions during the following winter (low rainfall; Table S2), which may have resulted in a superior advantage of more inbred individuals due to local maladaptation of outbred individuals (i.e., outbreeding depression; Marshall & Spalton, 2000; Marr, Keller, & Arcese, 2002). Outbred individuals resulted from immigrant–immigrant and local–immigrant crosses may be less fit than local (purebred) individuals if the latter are better adapted to their native habitats (e.g., García‐Navas, Ferrer, Sanz, & Ortego, 2014; Postma & van Noordwijk, 2005), a pattern that may only arise when benign environmental conditions relax the strength of selection on heterozygosity. This result is in agreement with that reported by Keller et al. (2002) in song sparrows (*Melospiza melodia*) as they found that inbred males produced fewer offspring in cooler years, but their productivity was similar in comparison with their outbred contemporaries after warm springs. Further, we have previously found that immigrant females, which represent the bulk of gene flow in blue tits, tend to be more heterozygous than residents in our study populations (García‐Navas, Ferrer, Sanz et al., 2014). More heterozygous immigrants may exhibit higher fecundity and viability than local individuals under stressful conditions but be in competitive disadvantage if more benign environmental circumstances make resident individuals superior due to their higher familiarity and/or adaptation to local conditions (Dias & Blondel, 1996). Thus, our results are in accordance with the finding of previous studies that the effects of heterozygosity on fitness are complex and positive and negative correlations can coexist due to the particular environmental context prevailing in different years within the same population (Lesbarrères et al., 2005; Chapman et al., 2009; see also Olano‐Marin, Mueller, & Kempenaers, 2011a).

Our analyses aimed to disentangle the underlying mechanism behind the observed HFC suggest that they are most likely explained by a general effect. We did not find heterozygosity at any particular marker, either putatively neutral or functional, to be significantly associated with fitness in any year. Single‐locus heterozygosity models did not improve the variance explained by multilocus heterozygosity models, indicating that genomewide heterozygosity is probably behind HFC in our study population (Szulkin et al., 2010). We neither found significant HFC when estimating heterozygosity from the subset of presumably functional loci, which is the group of markers most likely to show local or direct effects due to their location inside or flanking coding gene sequences that are being actively transcribed to RNA (Li et al., 2004; Olano‐Marin, Mueller, & Kempenaers, 2011a; Olano‐Marin, Mueller, & Kempenaers, 2011b). Thus, genomewide inbreeding seems to be the most plausible explanation for the observed HFC, a hypothesis that is also partially supported by the fact that we found evidence for ID (i.e., positive and significant *g* ^2^ values) in different study years (David et al., 2007; Szulkin et al., 2010; e.g., García‐Navas, Cáliz‐Campal et al., 2014).

### 4.2 Environmental harshness and strength of HFC

Several studies have found that the association between genetic diversity (or inbreeding) and different components of fitness become stronger under stressful conditions (Armbruster & Reed, 2005; Fox & Reed, 2011), but most evidence on this respect comes from experimental approaches and only a few studies have analyzed such interactions in wild populations (Forcada & Hoffman, 2014; Marr et al., 2006; Szulkin & Sheldon, 2007). Here, we quantified the relationship between heterozygosity and fitness along an environmental continuum, comprising harsh (wet–cool) and benign (dry–warm) years. Our results suggest that interannual variability in environmental harshness can affect the magnitude of HFC (Forcada & Hoffman, 2014; Keller et al., 2002). Among the studied environmental parameters, only accumulated rainfall was associated with selection differentials for heterozygosity (Figure 3). Severe rainfall events can lead to an increase in energy expenditure for flight and thermoregulation and a reduction in foraging efficiency, which can jeopardize substantially the fitness and survival prospects of songbirds (Avery & Krebs, 1984; Wilson et al., 2004). Accordingly, different studies have reported negative consequences of rainfall in terms of reproductive performance and survival (Arlettaz et al., 2010; Öberg et al., 2015), which highlights the important role of this meteorological variable as a selective agent in different life stages. The lack of association between temperature‐related parameters and the strength of HFC may be explained by the climate prevailing in the study area, which is characterized by mild winters with little or no snowfall (Tornero, 2003). In the Mediterranean region, winter conditions are less severe in comparison with temperate areas at higher latitudes, and thus, low temperatures are not likely to constitute an important stressor for birds in this region. On top of this, interannual variability in the number of days with extreme temperatures was moderately low (mean ± SD, TNZ: 246.0 ± 12.4; FDD: 32.2 ± 12.11; Table S2), which may explain that the strength of HFC respect to these variables has been stable over the study period irrespective of their relevance or not as a selective pressure. Unlike temperature, rainfall can vary markedly from year to year in the Mediterranean region (Blondel & Aronson, 1999). Accordingly, we found strong variability in accumulated precipitation within our small temporal series, which included dry and wet years (mean ± SD: 448.57 ± 164.33; Table S2). This fact may have increased variance in the strength of HFC among years with respect to this environmental variable and, thus, the chance that it is detected to be important by our analyses. Therefore, temporal and spatial variability in selective pressures may be an important factor explaining the direction and magnitude of the relationship between individual genetic diversity and fitness, which suggests the need to investigate HFC over a wide range of environmental conditions (Armbruster & Reed, 2005).

The overall positive selection differentials for heterozygosity observed for all markers (*S *=* *0.006) and the subsets of neutral (*S *=* *0.008) and putatively functional (*S *=* *0.001) loci (Table S2) seems to have resulted in an increase in mean population heterozygosity over the course of the study period (Figure 4). This increase in genetic diversity over time may have been in part reinforced by the moderate level of narrow‐sense heritability of heterozygosity (sensu Mitton, Schuster, Cothran, & deFries, 1993) found in our studied blue tit populations (*h* ^2^ = 0.3; García‐Navas et al., 2009) as well as a result of positive selection on heterozygosity for many other fitness‐related traits, including breeding performance (García‐Navas et al., 2009), resistance to parasites (Ferrer et al., 2014), and sexual selection (Ferrer et al., 2015; García‐Navas et al., 2009) (for other blue tit populations, see Foerster et al., 2003; Olano‐Marin, Mueller, & Kempenaers, 2011a; Olano‐Marin, Mueller, & Kempenaers, 2011b). Our results are thus in agreement with previous studies carried out in bottlenecked populations where selection against homozygotes is suggested as a possible mechanism for the maintenance or increase in heterozygosity over time (Bensch et al., 2006; Forcada & Hoffman, 2014; Kaeuffer et al., 2007).

### 4.3 Neutral *vs*. putatively functional markers

We found selection differentials to be correlated for the subsets of putatively functional and neutral markers, suggesting that different regions of the genome are subjected to similar evolutionary pressures (Figure 3). Also, heterozygosity estimated at putatively functional and neutral markers was correlated and, regardless of statistical significance, both subsets of loci showed similar trends of genetic diversity over the study years (Figure 4). Although HFC were predominately detected using all markers or the panel of neutral loci, the association between probability of interannual local recruitment and heterozygosity estimated at both subsets of markers showed similar trends in most years (see Table 2). Thus, our main results did not qualitatively differ between both subsets of loci and the observed differences in terms of statistical significance may be related to the lower power of functional markers to reflect genomewide inbreeding in comparison with neutral markers. Accordingly, all *g* ^2^ values were positive and many of them significant or marginally significant for all loci and the subset of neutral markers, whereas most *g* ^2^ values were not significant and often negative for the subset of putatively functional loci. These results are similar to those found in a previous study on blue tits comparing both kinds of markers and support the idea that neutral loci capture better the effects of genomewide inbreeding than functional markers (Ferrer et al., 2014; Olano‐Marin, Mueller, & Kempenaers, 2011b).

In comparison with previous studies using a similar approach, the similarities in the results obtained for the two subsets of markers are more remarkable than their differences (Ferrer et al., 2014, 2015; Olano‐Marin, Mueller, & Kempenaers, 2011a; Olano‐Marin, Mueller, & Kempenaers, 2011b). Such similarities may be in part attributable to the inherent difficulties of categorically classifying markers as putatively functional or neutral (Olano‐Marin, Mueller, & Kempenaers, 2011a; Olano‐Marin, Mueller, & Kempenaers, 2011b). On the one hand, it is expected that only a few (if any) of our putatively functional markers are actual functional variants on which selection is acting directly (Li et al., 2004). On the other hand, some of our functional markers may not be either affected by selection if the genes in which they lie are not themselves under strong selection (Ferrer et al., 2016). Conversely, some of our neutral markers can experience the effects of different forms of selection through LD with genes under selection located in nearby genomic regions. Thus, our two subsets of loci, classified considering whether they are located or not in genomic regions that are transcribed to RNA (Olano‐Marin, Mueller, & Kempenaers, 2011a; Olano‐Marin, Mueller, & Kempenaers, 2011b), may ultimately behave indistinguishably from each other. Collectively, our results suggest that certain characteristics present in the study populations (population substructure, high philopatry, and occurrence of incestuous matings; Ferrer et al., 2014, 2016; García‐Navas et al., 2009; Ortego, García‐Navas, Ferrer, & Sanz, 2011) may have contributed to generate ID, resulting in our two subsets of markers reflecting inbreeding, responding similarly to the effects of environmental‐driven selection (Figure 3), and showing comparable temporal trends of genetic diversity (Figure 4).

## 5 Conclusion

Overall, our study shows the importance of considering the environmental context in analyses about the consequences of individual genetic diversity on fitness. Future studies based on larger temporal series and employing more accurate estimates of fitness (e.g., lifetime reproductive success) and environmental harshness (e.g., food availability) may help to understand whether the strength of HFC in natural populations is influenced by stochastic, cyclic, or directional environmental changes (e.g., Forcada & Hoffman, 2014; Szulkin & Sheldon, 2007).

## Data Accessibility

All data used in this study are available in Dryad doi:10.5061/dryad.47pn4.

## Conflict of Interest

None declared.
