# Supplementary material for: The strength of the association between heterozygosity and probability of interannual local recruitment increases with environmental harshness in blue tits
Source: Ecol Evol. 2016 Nov 21;6(24):8857–69. doi: 10.1002/ece3.2591 (PMC5192745; doi:10.1002/ece3.2591)
Supplement: Supplementary file 1 [file ECE3-6-8857-s001.doc]

**Table S1.** Panel of 26 microsatellite loci used to genotype blue tits. The following information is given for each locus: chromosome location in the zebra finch genome, category (putatively neutral or functional), number of alleles (*K*), observed heterozygosity (*H*_O_), annealing temperature (T) and original reference or GenBank accession number.

| Locus | Chromosome | Category | *K* | *H*_O_ | T (ºC) | Reference |
| --- | --- | --- | --- | --- | --- | --- |
| Ase18 | 3 | Neutral | 13 | 0.88 | 60 | Richardson *et al*. 2000 |
| Mcyμ4 | 5 | Neutral | 13 | 0.84 | 50 | Double *et al*. 1997 |
| Pat-MP2-43 | 2 | Neutral | 7 | 0.45 | 59 | Otter *et al*. 1998 |
| Pca2 | Unassigned | Neutral | 9 | 0.73 | 60 | Dawson *et al*. 2000 |
| Pca3 | 4 | Neutral | 18 | 0.98 | 55 | Dawson *et al*. 2000 |
| Pca4 | 8 | Neutral | 9 | 0.69 | 60 | Dawson *et al*. 2000 |
| Pca7 | 1 | Neutral | 13 | 0.88 | 60 | Dawson *et al*. 2000 |
| Pca8 | 2 | Neutral | 26 | 0.88 | 53 | Dawson *et al*. 2000 |
| Pca9 | 7 | Neutral | 11 | 0.70 | 62 | Dawson *et al*. 2000 |
| Pdoμ5 | 4 | Neutral | 17 | 0.86 | 46 | Griffith *et al*. 1999 |
| PK11 | Unassigned | Neutral | 7 | 0.80 | 52 | GenBank Acc. no.: AF041465 |
| PK12 | 5 | Neutral | 14 | 0.85 | 62 | GenBank Acc. no.: AF041466 |
| Pocc1 | 7 | Neutral | 11 | 0.93 | 55 | Bensch *et al*. 1997 |
| Pocc6 | 2 | Neutral | 16 | 0.73 | 62 | Bensch *et al*. 1997 |
| CcaTgu7 | 2 | Functional | 5 | 0.59 | 55 | Olano-Marin *et al*. 2010 |
| CcaTgu8 | 2 | Functional | 3 | 0.52 | 63 | Olano-Marin *et al*. 2010 |
| CcaTgu11 | 3 | Functional | 6 | 0.71 | 60 | Olano-Marin *et al*. 2010 |
| CcaTgu14 | 5 | Functional | 13 | 0.73 | 55 | Olano-Marin *et al*. 2010 |
| CcaTgu15 | 5 | Functional | 4 | 0.82 | 60 | Olano-Marin *et al*. 2010 |
| CcaTgu19 | 10 | Functional | 21 | 0.96 | 60 | Olano-Marin *et al*. 2010 |
| CcaTgu28 | 23_random | Functional | 8 | 0.67 | 60 | Olano-Marin *et al*. 2010 |
| PiJ14 | 7 | Functional | 12 | 0.89 | 60 | Olano-Marin *et al*. 2010 |
| TG05-046 | 5 | Functional | 3 | 0.34 | 55 | Dawson *et al*. 2010 |
| TG05-053 | 5 | Functional | 8 | 0.74 | 55 | Dawson *et al*. 2010 |
| Tg13-017 | 13 | Functional | 14 | 0.84 | 60 | Dawson *et al*. 2010 |
| Tgu07 | 6 | Functional | 6 | 0.76 | 55 | Slate *et al*. 2007 |

**Table S2.** Estimates of environmental harshness (TNZ days: number of days with temperature ≥35 ºC or ≤ 15ºC; FDD: freezing-degree days, calculated as the number of days with temperature < 0 ºC; Precip.: accumulated precipitation in mm) used in this study (calculated from July 1st to March 31st), selection differentials (*S*) for heterozygosity and *g*^2^ values calculated for all markers and the subsets of neutral and putatively functional loci. *P*-values for *g*^2^ are indicated in parentheses and those statistically significant are in bold.

| Year | Environmental harshness | | |  | Selection differentials (*S*) | | |  | Identity disequilibrium (*g*^2^) | | |
| --- | --- | --- | --- | --- | --- | --- | --- | --- | --- | --- | --- |
|  | TNZ | FDD | Precip. |  | *S*_Total_ | *S*_Neutral_ | *S*_Functional_ |  | *g*^2^_Total_ | *g*^2^_Neutral_ | *g*^2^_Functional_ |
| 2008 | 239 | 15 | 351.8 |  | -0.002 | 0.006 | -0.012 |  | 0.003 (*P* = **0.028**) | 0.002 (*P* = 0.177) | -0.001 (*P* = 0.585) |
| 2009 | 267 | 32 | 600.9 |  | 0.013 | 0.018 | 0.008 |  | 0.006 (*P* = **0.001**) | 0.080 (*P* < **0.001**) | -0.006 (*P* =0.884) |
| 2010 | 237 | 22 | 505.3 |  | 0.005 | 0.002 | 0.008 |  | 0.001 (*P* = 0.151) | 0.078 (*P* < **0.001**) | -0.002 (*P* = 0.733) |
| 2011 | 234 | 46 | 238.2 |  | -0.022 | -0.029 | -0.022 |  | 0.002 (*P* = 0.100) | 0.003 (*P* = 0.087) | 0.004 (*P* = 0.239) |
| 2012 | 237 | 44 | 656.0 |  | 0.036 | 0.043 | 0.030 |  | 0.006 (*P* = **0.003**) | 0.004 (*P* = 0.077) | 0.017 (*P* = **0.010**) |
| 2013 | 238 | 34 | 339.2 |  | 0.003 | 0.007 | -0.006 |  | 0.001 (*P* = 0.285) | 0.001 (*P* = 0.423) | 0.002 (*P* = 0.302) |

**Table S3.** Model selection to assess the association between probability of inter-annual local recruitment (i.e. local recruitment from year *t* to year *t*+1) and genetic [A: heterozygosity estimated at all loci, *HL*_Total_] and non-genetic terms [B: locality; C: age; D: body condition; E: sex; F: fledging success; G: local/immigrant status]. Mating pair identity was included as random effect in all the models. Only best ranked equivalent models with ΔAICc ≤ 2 are shown.

| Model no. | Model | *K* | AIC_c_ | ∆AIC_c_ | ω*i* |
| --- | --- | --- | --- | --- | --- |
| (a) Local recruitment from 2008 to 2009 breeding season | | | | | |
| 1 | E+F+G | 5 | 140.79 | 0.00 | 0.17 |
| (b) Local recruitment from 2009 to 2010 breeding season | | | | | |
| 1 | A+B+D | 5 | 150.08 | 0.00 | 0.12 |
| 2 | B+D | 4 | 150.81 | 0.73 | 0.08 |
| 3 | A+B+D+G | 6 | 151.10 | 1.02 | 0.07 |
| 4 | A+B+D+F | 6 | 151.87 | 1.79 | 0.05 |
| 5 | B+D+G | 5 | 152.09 | 2.00 | 0.04 |
| (c) Local recruitment from 2010 to 2011 breeding season | | | | | |
| 1 | D | 3 | 158.09 | 0.00 | 0.10 |
| 2 | D+G | 4 | 158.72 | 0.64 | 0.07 |
| 3 | D+B | 4 | 159.25 | 1.17 | 0.06 |
| 4 | D+B+G | 5 | 159.64 | 1.55 | 0.05 |
| 5 | D+E | 4 | 159.84 | 1.76 | 0.04 |
| 6 | D+C | 4 | 159.87 | 1.78 | 0.04 |
| (d) Local recruitment from 2011 to 2012 breeding season | | | | | |
| 1 | A+D | 4 | 139.88 | 0.00 | 0.11 |
| 2 | A+B+D | 5 | 140.55 | 0.66 | 0.08 |
| 3 | A+D+G | 5 | 141.45 | 1.57 | 0.05 |
| 4 | A+C+D | 5 | 141.88 | 2.00 | 0.04 |
| (e) Local recruitment from 2012 to 2013 breeding season | | | | | |
| 1 | A+B+C+D | 6 | 81.88 | 0.00 | 0.21 |
| 2 | A+B+C+D+E | 7 | 83.36 | 1.47 | 0.10 |
| (f) Local recruitment from 2013 to 2014 breeding season | | | | | |
| 1 | D | 3 | 79.02 | 0.00 | 0.13 |
| 2 | D+F | 4 | 80.41 | 1.40 | 0.06 |
| 3 | C+D | 4 | 80.93 | 1.91 | 0.05 |

*K*, number of parameters in the model; AICc, corrected Akaike’s information criterion (AIC) value; ∆AICc, difference in AICc value from that of the strongest model; ω*i*, AICc weight.

**Table S4.** Model selection to assess the association between probability of inter-annual local recruitment (i.e. local recruitment from year *t* to year *t*+1) and genetic [A: heterozygosity estimated at the subset of neutral loci, *HL*_Neutral_; B: heterozygosity estimated at the subset of putatively functional loci, *HL*_Functional_] and non-genetic terms [C: locality; D: age; E: body condition; F: sex; G: fledging success; H: local/immigrant status]. Mating pair identity was included as random effect in all the models. Only best ranked equivalent models with ΔAICc ≤ 2 are shown.

| Model no. | Model | *K* | AIC_c_ | ∆AIC_c_ | ω*i* |
| --- | --- | --- | --- | --- | --- |
| (a) Local recruitment from 2008 to 2009 breeding season | | | | | |
| 1 | F+G+H | 5 | 149.79 | 0.00 | 0.15 |
| (b) Local recruitment from 2009 to 2010 breeding season | | | | | |
| 1 | A+C+E | 5 | 150.39 | 0.00 | 0.08 |
| 2 | C+E | 4 | 150.81 | 0.42 | 0.07 |
| 3 | A+C+E+H | 6 | 151.19 | 0.80 | 0.06 |
| 4 | A+C+E+G | 6 | 151.98 | 1.58 | 0.04 |
| 5 | B+C+E | 5 | 152.03 | 1.64 | 0.04 |
| 6 | C+E+H | 5 | 152.09 | 1.69 | 0.04 |
| 7 | C+E+G | 5 | 152.17 | 1.78 | 0.03 |
| 8 | A+B+C+E | 6 | 152.24 | 1.85 | 0.03 |
| (c) Local recruitment from 2010 to 2011 breeding season | | | | | |
| 1 | E | 3 | 157.38 | 0.00 | 0.07 |
| 2 | E+H | 4 | 157.87 | 0.49 | 0.06 |
| 3 | E+C | 4 | 158.63 | 1.26 | 0.04 |
| 4 | E+C+H | 5 | 158.63 | 1.50 | 0.04 |
| 5 | E+B | 4 | 158.88 | 1.57 | 0.03 |
| 6 | E+F | 4 | 158.95 | 1.66 | 0.03 |
| 7 | E+D | 4 | 159.04 | 1.80 | 0.03 |
| 8 | E+D+H | 5 | 159.18 | 1.94 | 0.03 |
| (d) Local recruitment from 2011 to 2012 breeding season | | | | | |
| 1 | A+B+C+E | 6 | 134.59 | 0.00 | 0.10 |
| 2 | A+B+E | 5 | 135.12 | 0.52 | 0.07 |
| 3 | A+B+C+D+E | 7 | 135.94 | 1.35 | 0.05 |
| (e) Local recruitment from 2012 to 2013 breeding season | | | | | |
| 1 | A+B+C+E+H | 7 | 79.46 | 0.00 | 0.13 |
| 2 | A+B+C+D+E | 7 | 79.76 | 0.31 | 0.11 |
| 3 | A+B+C+D+E+F | 8 | 80.74 | 1.28 | 0.07 |
| 4 | A+B+C+D+E+H | 8 | 80.96 | 1.50 | 0.06 |
| 5 | A+B+C+E+F+H | 8 | 81.30 | 1.84 | 0.05 |
| (f) Local recruitment from 2013 to 2014 breeding season | | | | | |
| 1 | A+B+H | 5 | 75.37 | 0.00 | 0.15 |

*K*, number of parameters in the model; AICc, corrected Akaike’s information criterion (AIC) value; ∆AICc, difference in AICc value from that of the strongest model; ω*i*, AICc weight.

**Table S5. Tests for the effects of single locus heterozygosity (SLH) on probability of inter-annual local recruitment (i.e. local recruitment from year *t* to year *t*+1). Table shows effect sizes, *Z* values and *P*-values. No test was significant after sequential Bonferroni correction.**

|  | From 2008 to 2009 | | | From 2009 to 2010 | | | From 2010 to 2011 | | | From 2011 to 2012 | | | | From 2012 to 2013 | | | From 2013 to 2014 | | |
| --- | --- | --- | --- | --- | --- | --- | --- | --- | --- | --- | --- | --- | --- | --- | --- | --- | --- | --- | --- |
| Locus | Effect size | *Z* | *P* | Effect size | *Z* | *P* | Effect size | *Z* | *P* | | Effect size | *Z* | *P* | Effect size | *Z* | *P* | Effect size | *Z* | *P* |
| *Ase18* | -0.056 | 0.34 | 0.564 | 0.144 | 2.43 | 0.122 | 0.024 | 0.06 | 0.802 | | 0.039 | 0.15 | 0.699 | 0.105 | 0.91 | 0.343 | -0.139 | 1.29 | 0.260 |
| *Mcyμ4* | 0.151 | 2.47 | 0.119 | 0.054 | 0.32 | 0.575 | 0.142 | 2.35 | 0.128 | | -0.027 | 0.08 | 0.783 | 0.046 | 0.17 | 0.680 | -0.014 | 0.01 | 0.904 |
| *Pat-MP2-43* | 0.036 | 0.14 | 0.712 | -0.040 | 0.18 | 0.674 | 0.056 | 0.36 | 0.547 | | -0.038 | 0.15 | 0.698 | -0.063 | 0.35 | 0.556 | -0.091 | 0.61 | 0.439 |
| *Pca2* | 0.021 | 0.05 | 0.831 | 0.110 | 1.37 | 0.244 | -0.070 | 0.57 | 0.450 | | -0.291 | 9.46 | 0.003 | -0.060 | 0.29 | 0.590 | 0.101 | 0.71 | 0.404 |
| *Pca3* | 0.075 | 0.60 | 0.442 | 0.053 | 0.32 | 0.572 | 0.110 | 1.43 | 0.235 | | -0.034 | 0.12 | 0.728 | 0.101 | 0.92 | 0.340 | -0.135 | 1.36 | 0.247 |
| *Pca4* | 0.104 | 1.14 | 0.288 | -0.057 | 0.36 | 0.549 | 0.068 | 0.54 | 0.464 | | -0.242 | 6.45 | 0.013 | 0.121 | 1.27 | 0.263 | -0.014 | 0.01 | 0.904 |
| *Pca7* | 0.048 | 0.24 | 0.624 | 0.078 | 0.67 | 0.416 | -0.075 | 0.66 | 0.417 | | -0.099 | 1.03 | 0.313 | 0.144 | 1.77 | 0.186 | 0.026 | 0.05 | 0.825 |
| *Pca8* | -0.185 | 3.69 | 0.057 | 0.085 | 0.80 | 0.374 | 0.004 | 0.00 | 0.968 | | -0.042 | 0.18 | 0.674 | 0.032 | 0.08 | 0.771 | -0.142 | 1.49 | 0.226 |
| *Pca9* | 0.030 | 0.10 | 0.758 | 0.078 | 0.70 | 0.403 | -0.084 | 0.82 | 0.366 | | -0.107 | 1.22 | 0.272 | -0.020 | 0.03 | 0.853 | 0.070 | 0.36 | 0.550 |
| *Pdoμ5* | 0.016 | 0.03 | 0.874 | 0.013 | 0.02 | 0.897 | 0.075 | 0.59 | 0.445 | | 0.039 | 0.14 | 0.711 | -0.049 | 0.18 | 0.675 | 0.139 | 1.35 | 0.250 |
| *PK11* | -0.002 | 0.00 | 0.980 | -0.079 | 0.68 | 0.410 | 0.056 | 0.35 | 0.553 | | -0.051 | 0.28 | 0.600 | 0.123 | 1.27 | 0.263 | 0.196 | 2.91 | 0.092 |
| *PK12* | -0.089 | 0.86 | 0.356 | 0.178 | 3.70 | 0.057 | -0.043 | 0.21 | 0.646 | | -0.034 | 0.12 | 0.728 | -0.005 | 0.00 | 0.965 | 0.048 | 0.17 | 0.686 |
| *Pocc1* | 0.131 | 1.82 | 0.180 | 0.105 | 1.23 | 0.270 | 0.079 | 0.73 | 0.395 | | 0.003 | 0.00 | 0.975 | 0.137 | 1.68 | 0.198 | 0.150 | 1.67 | 0.201 |
| *Pocc6* | -0.142 | 2.20 | 0.141 | -0.143 | 2.37 | 0.127 | -0.170 | 3.48 | 0.065 | | 0.103 | 1.13 | 0.290 | 0.129 | 1.47 | 0.229 | -0.101 | 0.74 | 0.393 |
| *CcaTgu7* | -0.077 | 0.60 | 0.442 | -0.011 | 0.01 | 0.916 | 0.079 | 0.68 | 0.411 | | -0.121 | 1.38 | 0.244 | -0.056 | 0.24 | 0.623 | -0.160 | 1.79 | 0.186 |
| *CcaTgu8* | -0.136 | 1.86 | 0.175 | -0.068 | 0.48 | 0.489 | 0.072 | 0.56 | 0.455 | | -0.078 | 0.58 | 0.448 | 0.084 | 0.49 | 0.484 | 0.042 | 0.12 | 0.732 |
| *CcaTgu11* | -0.090 | 0.87 | 0.352 | 0.041 | 0.18 | 0.673 | 0.103 | 1.23 | 0.270 | | 0.042 | 0.17 | 0.679 | -0.071 | 0.36 | 0.551 | 0.079 | 0.44 | 0.510 |
| *CcaTgu14* | -0.034 | 0.13 | 0.723 | 0.149 | 2.45 | 0.121 | -0.015 | 0.02 | 0.877 | | 0.035 | 0.12 | 0.729 | 0.131 | 1.32 | 0.253 | 0.074 | 0.38 | 0.538 |
| *CcaTgu15* | 0.10 | 1.23 | 0.269 | 0.114 | 1.39 | 0.241 | 0.095 | 1.03 | 0.312 | | 0.094 | 0.88 | 0.350 | -0.051 | 0.22 | 0.640 | 0.048 | 0.16 | 0.688 |
| *CcaTgu19* | -0.044 | 0.20 | 0.652 | 0.024 | 0.06 | 0.806 | -0.188 | 4.16 | 0.044 | | -0.124 | 1.52 | 0.220 | -0.130 | 1.20 | 0.277 | 0.075 | 0.39 | 0.532 |
| *CcaTgu28* | -0.087 | 0.81 | 0.371 | 0.006 | 0.00 | 0.949 | 0.002 | 0.00 | 0.980 | | 0.008 | 0.01 | 0.940 | 0.194 | 2.92 | 0.092 | -0.013 | 0.01 | 0.922 |
| *PiJ14* | 0.064 | 0.45 | 0.506 | -0.006 | 0.00 | 0.953 | -0.148 | 2.61 | 0.109 | | 0.037 | 0.14 | 0.711 | 0.025 | 0.05 | 0.823 | 0.197 | 2.90 | 0.093 |
| *TG05-046* | 0.115 | 1.37 | 0.245 | -0.076 | 0.61 | 0.435 | 0.022 | 0.05 | 0.818 | | 0.055 | 0.29 | 0.594 | 0.076 | 0.46 | 0.500 | 0.071 | 0.35 | 0.555 |
| *TG05-053* | 0.010 | 0.01 | 0.922 | -0.019 | 0.04 | 0.845 | 0.169 | 3.34 | 0.070 | | -0.110 | 1.23 | 0.271 | -0.050 | 0.20 | 0.654 | -0.115 | 0.96 | 0.331 |
| *Tg13-017* | 0.037 | 0.14 | 0.713 | 0.046 | 0.21 | 0.650 | 0.099 | 1.07 | 0.304 | | -0.052 | 0.25 | 0.618 | 0.051 | 0.21 | 0.646 | -0.284 | 5.97 | 0.017 |
| *Tgu07* | -0.115 | 1.43 | 0.235 | -0.103 | 1.18 | 0.279 | -0.021 | 0.05 | 0.821 | | -0.035 | 0.12 | 0.729 | 0.120 | 1.04 | 0.310 | -0.011 | 0.01 | 0.924 |

**Supplementary References**

Bensch, S., Price, T. & Kohn, J. (1997) Isolation and characterization of microsatellite loci in a *Phylloscopus* warbler. *Molecular Ecology*, **6**, 91−92.

Dawson, D.A., Hanotte, O., Greig, C., Stewart, I.R.K. & Burke, T. (2000) Polymorphic microsatellites in the blue tit Parus caeruleus and their cross-species utility in 20 songbird families. *Molecular Ecology*, **9**, 1941–1944.

Dawson, D.A., Horsburgh, G.J., Küpper, C., Stewart, I.R.K., Ball, A.D., Durrant, K.L., *et al*. (2010) New methods to identify conserved microsatellite loci and develop primer sets of high cross-species utility - as demonstrated for birds. *Molecular Ecology Resources*, **10**, 475−494.

Double, M.C., Dawson, D., Burke, T. & Cockburn, A. (1997) Finding the fathers in the least faithful bird: A microsatellite-based genotyping system for the superb fairy-wren *Malurus cyaneus*. *Molecular Ecology*, **6**, 691−693.

Griffith, S.C., Stewart, I.R.K., Dawson, D.A., Owens, I.P.F. & Burke, T. (1999) Contrasting levels of extra-pair paternity in mainland and island populations of the house sparrow (*Passer domesticus*): is there an “island effect”? *Biological Journal of Linnean Society*, **68**, 303−316.

Olano-Marin, J., Dawson, D.A., Girg, A., Hansson, B., Ljungqvist, M., Kempenaers, B. & Mueller, J.C. (2010) A genome-wide set of 106 microsatellite markers for the blue tit (*Cyanistes caeruleus*). *Molecular Ecology Resources*, **10**, 516−532.

Otter, K., Ratcliffe, L., Michaud, D. &Boag, P.T. (1998) Do female black-capped chickadees prefer high-ranking males as extra-pair partners? *Behaviour Ecology Sociobiology*, **43**, 25−36.

Richardson, D.S., Jury, F.L., Dawson, D.A., Salgueiro, P., Komdeur, J. & Burke, T. (2000) Fifty Seychelles warbler (*Acrocephalus sechellensis*) microsatellite loci polymorphic in Sylviidae species and their cross-species amplification in other passerine birds. *Molecular Ecology*, **9**, 2226−2231.

Slate, J., Hale, M. & Birkhead, T.R. (2007) Simple sequence repeats in zebra finch (*Taeniopygia guttata*) expressed sequence tags: a new resource for evolutionary genetic studies of passerines. *BMC Genomics*, **8**, 52.
